# Acu-TENS Reduces Breathlessness during Exercise in People with Chronic Obstructive Pulmonary Disease 

**DOI:** 10.1155/2017/3649257

**Published:** 2017-02-20

**Authors:** Shirley P. C. Ngai, Lissa M. Spencer, Alice Y. M. Jones, Jennifer A. Alison

**Affiliations:** ^1^Department of Rehabilitation Sciences, The Hong Kong Polytechnic University, Kowloon, Hong Kong, China; ^2^Department of Physiotherapy, Royal Prince Alfred Hospital, Sydney, NSW, Australia; ^3^Discipline of Physiotherapy, The University of Sydney, Sydney, NSW, Australia

## Abstract

*Background.* Exertional dyspnoea limits level of physical activity in people with Chronic Obstructive Pulmonary Disease (COPD). This randomized, double-blinded, crossover study evaluated the effect of Acu-TENS, application of Transcutaneous Electrical Nerve Stimulation on acupoints, on breathlessness during exercise in people with COPD.* Methods.* Twenty-one participants, mean% predicted FEV_1_  50 ± 21%, attended assessment followed by two intervention days, one week apart. On each intervention day, participants performed two endurance shuttle walk tests (ESWT) (Walk 1 and Walk 2). Walk 1 was performed without intervention and Walk 2 was performed with either Acu-TENS or Sham-TENS, in random order, for 45 minutes before and during Walk 2. Duration of each ESWT and dyspnoea score at isotime of Walk 1 and Walk 2 on each intervention day were compared. Between-group differences in ESWT duration and isotime dyspnoea were also compared.* Results.* At isotime of Walk 1 and Walk 2, Acu-TENS showed significant reduction in dyspnoea of −0.8 point (95% CI −0.2 to −1.4) but not in Sham-TENS [0.1 point (95% CI −0.4 to 0.6)]. Compared to Sham-TENS, Acu-TENS showed significant reduction in dyspnoea of −0.9 point (95% CI −0.2 to −1.6) while no between-group significance was found in ESWT duration.* Conclusion.* Acu-TENS alleviated dyspnoea during walking in people with COPD but did not increase walking duration.

## 1. Introduction

Exertional dyspnoea is one of the major disabling symptoms of people with Chronic Obstructive Pulmonary Disease (COPD), limiting physical activity, and even activities of daily living. Muscle deconditioning, as a consequence of decreased functional activities, further reduces exercise tolerance.

Application of Transcutaneous Electrical Nerve Simulation on acupoints (Acu-TENS) is an integrative intervention merging Transcutaneous Electrical Nerve Stimulation (TENS), a western treatment modality, with acupuncture, a common Chinese therapeutic approach to disease management. Unlike traditional acupuncture which is invasive, as insertion of a needle into the acupuncture point is necessary for stimulation, Acu-TENS is a noninvasive intervention, which induces acupoint therapeutic effects through application of surface electrodes over the acupuncture points. A single session of Acu-TENS has been shown to alleviate dyspnoea at rest in people with COPD in a stable condition [1–3] and during an exacerbation [4]. However, the effect of Acu-TENS on breathlessness during exercise and its influence on exercise capacity in people with COPD has not been explored.

We hypothesized that applying TENS to specific acupoints for breathlessness (EX-B1/Dingchuan) would relieve dyspnoea during exercise and would result in increased exercise duration when compared to Sham-TENS. Thus, the aim of the study was to evaluate the effect of Acu-TENS compared to Sham-TENS on dyspnoea during exercise and on exercise duration in people with COPD in a stable condition.

## 2. Methods

### 2.1. Participants

Individuals diagnosed with COPD at GOLD I to GOLD IV defined by the Global Initiative for Chronic Obstructive Lung Disease (GOLD) [5] were recruited and assessed for eligibility. Participants were excluded if any of the following criteria applied: (1) acute exacerbation of COPD within the last 4 weeks; (2) significant comorbidity including symptomatic cardiovascular disease or musculoskeletal disorder or neurological disorder that could limit exercise performance; (3) supplemental oxygen required during exercise or use of a walking aid; (4) allergic to gel; and (5) cognitive disorder. The study was conducted in accordance with the Declaration of Helsinki [6]. Ethics approval was granted by the Human Ethics Review Committee of the hospital (RPAH zone, Sydney, Australia). Written informed consent was obtained from all participants prior to the study (trial registration: ANZCTRN12612000693820).

### 2.2. Study Design

**Figure 1:**
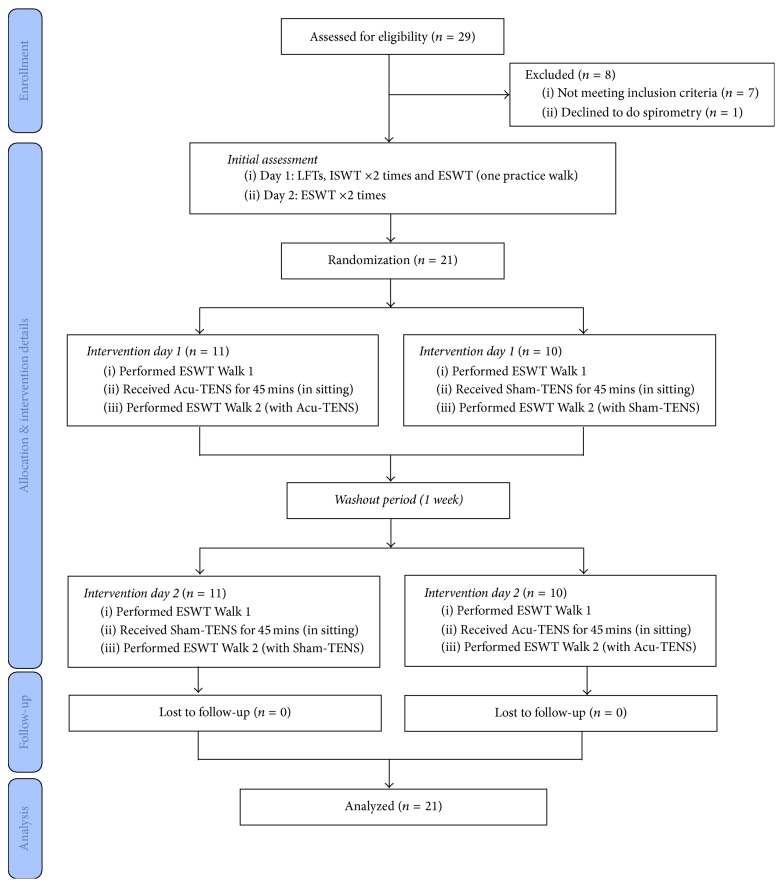
Study flow chart.

The study was a randomized, controlled crossover study with both assessor and participants blinding, conducted at Royal Prince Alfred Hospital, Sydney, Australia. Recruited participants attended for four visits. All participants continued their usual medications before each visit and were scheduled at the same time on each visit. The initial assessments occurred over two days within one week, followed by two intervention days (intervention days 1 and 2) at least one week apart to allow for a washout period (Figure 1). For the initial assessments, participants performed lung function tests consisting of spirometry (EasyOne, ndd Medical Technologies Inc., Andover, MA, USA) and lung volumes measured by body plethysmography (*V*max Autobox V62J, SensorMedics, Yorba Linda, CA) following standardized procedures [7] and compared to normative values [8]. In addition, participants performed two incremental shuttle walk tests (ISWT) and one practice of the endurance shuttle walk test (ESWT) for familiarization. Participants completed a short questionnaire to determine their previous experience of acupuncture and TENS and their opinions on safety and effectiveness of Acu-TENS. On another day, participants performed two ESWTs to ensure complete familiarization and to exclude a learning effect prior to the intervention days. All walk tests were separated by 30-minute rest.

**Figure 2:**
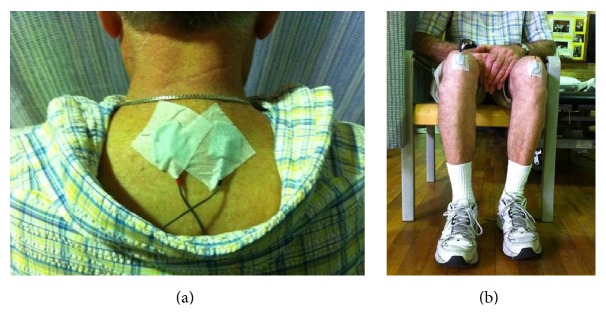
Points chosen for Acu-TENS and Sham-TENS. (a) Acu-TENS: application of TENS on acupuncture points, EX-B1 (Dingchuan). (b) Sham-TENS: application of TENS on nonacupuncture points, patella.

On each intervention day, participants performed two ESWTs (Walk 1 and Walk 2). Walk 1 was performed without any intervention. Walk 2 on each intervention day was preceded by either Acu-TENS (Figure 2(a)) or Sham-TENS (Figure 2(b)) for 45 minutes which continued during Walk 2. The order of Acu-TENS or Sham-TENS on intervention days 1 and 2 was randomized by a computer software (Randomization Allocation Software, version 1.0, Isfahan University of Medical Sciences, Iran) and the randomization sequence was concealed in an opaque envelopes by an investigator who was not involved in any of the assessments or intervention procedures. The study flow chart is shown in Figure 1.

### 2.3. Incremental Shuttle Walk Test (ISWT)

The ISWT was performed based on standardized protocol [9]. During the ISWT, participants were asked to walk back and forth along a 10-metre path following the speed preset by a compact disc (CD) player, with speed increased every minute. The test was terminated if participants failed to keep up with the speed or stopped because of symptoms. Two ISWTs, at least 30 minutes apart, were performed to attain an accurate prediction of peak exercise capacity [10]. The better distance covered in the two tests was used to calculate the predicted peak oxygen consumption (*V*O_2peak_). Eighty-five percent of *V*O_2peak_ was used to calculate the corresponding speed for the subsequent ESWT [11].

### 2.4. Endurance Shuttle Walk Test (ESWT)

The ESWT was conducted with participants walking back and forth along a 10-metre track at a speed calculated from the ISWT (i.e., speed at 85% of predicted peak oxygen consumption) [11]. At the beginning of the ESWT, participants walked at a slow preset speed for two minutes as warm-up and then walked at the walking speed calculated from the ISWT. The test terminated when the participants either failed to keep up with the speed or stopped because of symptoms.

During the ISWT and ESWT, heart rate (HR) was measured by a heart rate monitor (Polar RS800CX, Finland), oxygen saturation (SpO_2_), by a pulse oximeter (Rad-5v™, Masimo Corporation, USA), and level of dyspnoea, by the 0–10-category-ratio Borg scale [12]. All measurements were recorded before the test, at the end of every minute during and immediately after test completion.

### 2.5. Intervention

#### 2.5.1. Acu-TENS or Sham-TENS

**Figure 3:**
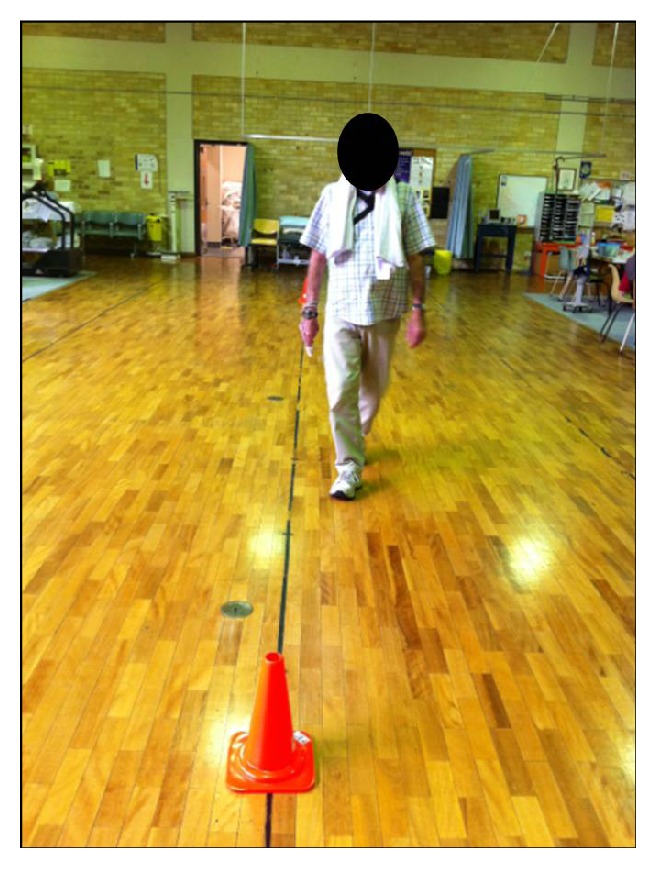
One participant performing the 2nd ESWT with Acu-TENS/Sham-TENS during the intervention day. Note: to ensure blinding of assessor to application position the leads for both applications protruded at the waist and all participants wore a towel around the neck and long pants for both applications.

Acu-TENS was applied with two electrodes from the TENS machine (ITO ES320, ITO Ltd., Japan; weight of machine: 120 grams) placed over the acupoints for alleviating breathlessness (EX-B1/Dingchuan), located at 0.5 cun lateral to the lower border of the 7th cervical vertebrae. “Cun” is defined as the distance between medial creases of the interphalangeal joint of the middle finger of the individual participant (Figure 2(a)). For stimulation with Sham-TENS, the two electrodes were applied over each patella (nonacupoint) (Figure 2(b)) [2] which did not interfere or restrict any movement. The position of the electrodes for TENS was concealed from the assessor by a towel covering the neck and the participants wearing long pants (Figure 3). For both Acu-TENS and Sham-TENS, the TENS machine was preset at 2 Hz burst mode with a pulse width of 200 *μ*s for 45 minutes [2–4]. The intensity of stimulation was adjusted to the maximally tolerable level but short of pain. After the first ESWT (Walk 1) on each of the intervention days, participants received either Acu-TENS or Sham-TENS for 45 minutes prior to and during the second ESWT (Walk 2).

### 2.6. Outcome Measures

The primary outcome measures were dyspnoea (measured by modified Borg scale [12]) at isotime between Walk 1 and Walk 2 and dyspnoea at isotime between Acu-TENS and Sham-TENS. Isotime was defined as the duration of the shorter ESWT (to the nearest minute) of Walk 1 and Walk 2 on each of the intervention days and when Acu-TENS was directly compared to Sham-TENS.

The secondary outcome measure was the change in ESWT duration between Walk 1 and Walk 2 on intervention days.

### 2.7. Statistical Analysis

Based on a medium effect size (*f* = 0.35), a power of 0.8 and *α* of 0.05 with repeated measure ANOVA, a minimum sample of 20 participants was required (G*∗*Power 3.1.9.2, Universitat Kiel, Germany). All data are presented as mean and standard deviation (SD). Level of dyspnoea measured at isotime and the duration of the two ESWTs (within group effect: Walk 1 and Walk 2) recorded on each of the intervention days and intervention effect (between-group effect: Acu-TENS and Sham-TENS) were compared by repeated measure ANOVA with subsequent analysis if main or interaction effects were significant. Association of change in dyspnoea and the change in walking distance was examined using Pearson's correlation with significance level set at *p* < 0.05. Data analysis was conducted by using IBM SPSS (version 23.0, IBM Corporation, Armonk, NY).

## 3. Results

### 3.1. Demographic Data

Twenty-nine participants were recruited for the study; seven were excluded because of the presence of either neurological condition (*n* = 1) or musculoskeletal pain, including osteoarthritic knees and hip (*n* = 6) that may have affected exercise performance and one declined to perform lung function tests (*n* = 1). Twenty-one participants (20 Caucasian and one Chinese ethnicity), aged (mean ± SD) 70 ± 6 years, completed the study. The mean time since diagnosis of COPD was 8 ± 1 years with a mean% predicted forced expiratory volume in one second (FEV_1_) of 50 ± 21%. Nineteen participants were ex-smokers and two were current smokers, with a mean pack-year of 56 ± 28 pack-years for the group. Demographic characteristics are summarized in Table 1. No adverse event was reported during the study period. Majority of the participants (*n* = 15) reported dyspnoea to be the limiting factor at the end of ESWT while the remaining 28.5% of the participants (*n* = 5) reported that both dyspnoea and leg fatigue to be the main factors that refrained them from continuing ESWT.

Eighty-one percent of the participants reported no previous experience of acupuncture and all participants were TENS naive. Prior to any intervention, the questionnaire revealed that most of the participants (91%) thought that Acu-TENS would be safe and 43% thought it would reduce breathlessness during exercise.

### 3.2. Primary Outcome Measure

Repeated measure ANOVA revealed that there were significant interaction effects between time and intervention in dyspnoea at isotime (*F* = 7.9, *p* = 0.01). When Walk 1 (no intervention) was compared to Walk 2 (Acu-TENS) at isotime there was a significant reduction in dyspnoea score due to Acu-TENS (mean difference −0.8 ± 1.3 points (95% CI −0.2 to −1.4, *p* = 0.01)). When Walk 1 (no intervention) was compared to Walk 2 (Sham-TENS) no difference in dyspnoea score was observed (mean difference 0.1 ± 1.1 points, 95% CI −0.4 to 0.6, *p* = 0.62).

A direct comparison between Acu-TENS and Sham-TENS at isotime showed a significant reduction in dyspnoea of −0.9 points (95% CI −1.6 to −0.2, *p* = 0.01) in favour of Acu-TENS. A subgroup analysis of participants with dyspnoea ≥ 4 at end of the ISWT (*n* = 12) showed a significant reduction in dyspnoea with Acu-TENS compared to Sham-TENS of −1 point (95% CI −1.7 to −0.3, *p* = 0.01), while no significant change was observed in participants with dyspnoea < 4 points (*n* = 9) (mean difference −0.8 points, 95% CI −2.3 to 0.7, *p* = 0.26).

### 3.3. Secondary Outcome Measure

No significant differences were found in time and group interaction in the duration of Walk 1 and Walk 2 on intervention days 1 and 2 (*F* = 0.12, *p* = 0.74) (Table 2).

### 3.4. Association between Change in Dyspnoea and Change in Walking Distance

A decrease in dyspnoea at isotime in the ESWTs during Acu-TENS was found to be moderately correlated with the increase in ESWT duration (*r* = −0.56, *p* = 0.01).

## 4. Discussion

This study showed that Acu-TENS significantly reduced the level of dyspnoea at exercise isotime compared to no intervention and compared to Sham-TENS in people with stable COPD. The between-group difference in dyspnoea score was 0.9 points, and for those participants with higher levels of dyspnoea at peak exercise (i.e., ≥4 on the ISWT) the reduction in dyspnoea at isotime on the ESWT was 1.0 point. A one-point change in dyspnoea score has been reported to be the minimally clinically important difference in response to acute administration of oxygen supplementation or bronchodilator therapy in people with COPD [13].

Opioid release induced by TENS, similar to acupuncture, is frequency dependent [14]. Our study used a TENS protocol with a preset frequency of 2 Hz and an intensity of maximal tolerable but short of pain because low frequency high intensity TENS has been reported to induce the release of *β* endorphin, endomorphin, and met-enkephalin levels [14]. Upon stimulation, endorphin and encephalin preferentially act on *μ*- and *δ*-opioid receptors [15, 16] leading to a reduction in respiratory frequency [17, 18] and relaxation of airway smooth muscle via beta adrenoceptor in animal models [19]. Previous studies showed that Acu-TENS could trigger the release of *β* endorphin in people with COPD and that the changes were associated with an increase in FEV_1_, [3] decrease in respiratory rate and reduction in oxygen desaturation after 6-minute walk test in people with COPD [2]. The increase in *β* endorphins was found in the Acu-TENS group but not in the placebo comparators, that is, application of TENS to nonacupuncture (Sham-TENS) or application to acupoints without electrical stimulation (Placebo-TENS), which indicated that the response was due to the stimulation of acupuncture points rather than merely an electricity effect [2]. These outcomes were in accordance with the current findings that Acu-TENS reduced the level of dyspnoea during exercise at an equivalent work rate (isotime) when compared with Sham-TENS. Subgroup analysis showed that the reduction in dyspnoea at isotime was greater in those participants who reported more breathlessness (dyspnoea ≥ 4) at peak exercise.

Although Acu-TENS reduced dyspnoea at isotime, there was no significant difference in ESWT duration when Acu-TENS was compared either with no intervention (within group comparison) or with Sham-TENS (between-group comparison). In this study, 28.5% of participants reported both dyspnoea and leg fatigue to be the limiting factors to exercise. In particular, those who reported more breathlessness (dyspnoea ≥ 4 at peak exercise), about 42% (5 out of 12) of them reported both dyspnoea and leg fatigue to be the main factors that refrained them from continuing ESWT. While dyspnoea is an important contributor to exercise intolerance, the ESWT duration may also have been influenced by other factors such as deconditioning associated muscle weakness and fatigue. Thus one session of Acu-TENS on acupoints specific to alleviate breathlessness may not have been adequate to increase endurance capacity. Interestingly, a moderate correlation (*r* = −0.56, *p* = 0.01) between decrease in dyspnoea at isotime and increased ESWT duration was observed. Future studies examining the effects of Acu-TENS during exercise training are warranted to determine whether the reduction in dyspnoea would enable higher exercise intensities and/or longer exercise durations, thereby maximizing the training effect of rehabilitation. In addition, as Acu-TENS has the potential to be self-administered by patients and/or caregivers, future studies could evaluate the effectiveness of Acu-TENS to reduce dyspnoea during daily activities.

One limitation of this study was that the level of *β* endorphin was not measured. Such measurements would have helped to elucidate mechanisms of dyspnoea reduction.

## 5. Conclusion

In summary, this study demonstrated that Acu-TENS reduced dyspnoea during walking in people with COPD, particularly in those who had more severe dyspnoea at peak exercise. Further investigation is required to determine whether the reduction in dyspnoea during Acu-TENS could enable higher exercise intensity during training or reductions in dyspnoea during daily activities.

## Figures and Tables

**Table 1:** Characteristics of participants.

	Mean ± SD
Age, years	70 ± 6
Male, number (%)	11 (52%)
Height, m	1.66 ± 0.08
Weight, kg	71 ± 20
BMI, kg/m^2^	25.5 ± 6.5
Cun, cm	2.1 ± 0.2
Pack years, years	56 ± 28
GOLD I/II/III/IV, (%)	10/47/29/14
FEV_1_, % pred	50 ± 21
FVC, % pred	83 ± 22
FEV_1_/FVC ratio	0.48 ± 0.14
TLC, % pred	118 ± 20
FRC, % pred	146 ± 34
RV, % pred	167 ± 60
SGRQ, symptoms	46 ± 22
SGRQ, activity	62 ± 14
SGRQ, impact	29 ± 15
SGRQ, total	43 ± 14

Data are presented as mean ± standard deviation (SD). BMI = Body Mass Index; cun refers to the basic measurement unit for locating acupuncture points, that is, distance between the medical creases of the interphalangeal joint of middle finger of participants; FEV_1_ = forced expiratory volume in 1 second; FVC = forced vital capacity; FRC = functional residual capacity; RV = residual volume; SGRQ = St. George's Respiratory Questionnaire.

**Table 2:** Comparison of outcome measures from Walk 1 (no intervention) and Walk 2 (either Acu-TENS or Sham-TENS) on each of the intervention days.

	Acu-TENS (*n* = 21)			Sham-TENS (*n* = 21)			Between-group difference (Acu-TENS minus Sham-TENS) (95% CI) *p* value	Repeated measure ANOVA	
	Walk 1	Walk 2	Within-group difference (95% CI) *p* value	Walk 1	Walk 2	Within-group difference (95% CI) *p* value		*F*	*p* value
*Dyspnoea*									
At isotime (points)	5.0 ± 2.1	4.2 ± 1.8	−0.8 (−0.2 to −1.4) *p* = 0.01	4.4 ± 2.2	4.5 ± 2.5	0.1 (−0.4 to 0.6) *p* = 0.62	−0.9 (−0.2 to −1.6) *p* = 0.01	7.9	0.01
End of ESWT (points)	5.6 ± 1.9	5.1 ± 2.0	−0.5 (−0.1 to −0.8) *p* = 0.01	5.2 ± 2.3	5.1 ± 2.4	−0.1 (−0.4 to 0.3) *p* = 0.67	−0.4 (−0.1 to −0.8) *p* = 0.03	5.4	0.03
*ESWT*									
Time (seconds)	371 ± 207	413 ± 240	42 (−8 to 92) *p* = 0.10	363 ± 198	399 ± 191	36 (−18 to 90) *p* = 0.18	6 (−58 to 69) *p* = 0.86	0.03	0.86
Distance (metres)	494 ± 352	543 ± 368	49 (−18 to 116) *p* = 0.14	488 ± 339	526 ± 308	37 (−18 to 93) *p* = 0.17	21 (−62 to 104) *p* = 0.60	0.12	0.74

Data are presented as mean ± SD. ESWT = endurance shuttle walk test.
